# Combination of ifosfamide and etoposide as a salvage regimen for previously treated soft tissue sarcomas: a retrospective single centre study

**DOI:** 10.3332/ecancer.2022.1363

**Published:** 2022-03-03

**Authors:** Luiz Fernando Ribeiro, Fernando Augusto Batista Campos, Celso Abdon Mello

**Affiliations:** Departament of Medical Oncology, A C Camargo Cancer Center, São Paulo 01509-010, SP, Brazil

**Keywords:** sarcoma, treatment, prognosis, chemotherapy

## Abstract

**Introduction:**

Systemic treatment for metastatic soft tissue sarcoma (STS) results in modest activity in second and further lines. The aim of this study was to evaluate the efficacy of ifosfamide and etoposide (IE) as a salvage regimen for patients with metastatic STS.

**Methods:**

A retrospective, single centre study included patients with STS treated with IE from 2010 to 2018. The primary endpoint was progression-free survival (PFS). Secondary endpoints were toxicity, response rate (RR) and overall survival (OS). Survival was estimated by the Kaplan–Meier method and log-rank test used to compare the groups.

**Results:**

A total of 33 patients were identified, median age was 43 years, 60% were female, 12 had leiomyosarcoma. IE was used in second line in 51.5% and in >third line in 30.3% of patients. Median number of cycles was four and treatment discontinuation due to grade 3/4 toxicity occurred in 30.3%. The objective RR was 9% and the disease control rate was 60.6%. Median PFS was 4 months (95% CI, 2.1–5.9) and the median OS was 15 months (95% CI, 7.1–22.9). In the univariate analysis, smoking history, line of therapy and prior response to previous chemotherapy were prognostic factors for PFS.

**Conclusion:**

IE showed activity in previously treated STS, but with a non-negligible toxicity profile, worse than that with other available therapies. The use of the IE combination is not supported by our findings outside a clinical trial for soft part sarcomas.

## Introduction

Soft tissue sarcomas (STS) are a group of rare and heterogenous neoplasms that represents around 1% of all solid tumours in adults [1]. Currently, the World Health Organization recognises more than 100 histological subtypes of sarcomas [2]. The main treatment for localised disease is surgery [3]. Perioperative radiation therapy and chemotherapy are employed for high-risk patients [3, 4]. Despite the increase in local control rates, almost 50% of the patients evolves with distant disease relapse [5]. Many agents have been incorporated in the palliative treatment scenario in the past years. However, the median overall survival (OS) of patients with metastatic disease does not surpass 20 months in the most recent trials [6].

Doxorubicin continues to be the standard treatment for most histologies in the first line [3, 7]. The combination of doxorubicin with other agents did not proved to increase OS, despite the increment in the response rate (RR) [8, 9]. In the second line, there are many approved agents such as gemcitabine [10], docetaxel [11], trabectedin [12], dacarbazine [11], eribulin [13] for the adipocytic and pazopanib [14] for the non-adipocytic sarcomas. Nevertheless, the progression-free survival (PFS) and RRs of second- and third-line agents are very limited [10–14], showing that a more precise strategy needs to be adopted when clinical trials are designed, specially taking in account the specific histologies.

In many countries, important agents for the treatment of metastatic STS are not routinely available, such as trabectedin, pazopanib and eribulin [7]. As a result, alternative regimens are used in daily practice for selected young patients, notably those with good performance status, that present disease progression. Ifosfamide combined with etoposide (IE) has substantial activity in a wide range of malignancies and is active against Ewing sarcoma [15] and osteosarcoma [16]. However, there is limited evidence about the efficacy of IE in adult patients with STS [17, 18]. In Brazil, for instance, in the private sector, there is a two-step system for approval of oral drugs to treat cancer. Pazopanib was approved by ANVISA (National Agency) but it was not incorporated in the list of drugs with reimbursement by the health care insurance companies. On the other hand, in the public system, the resources released to treat patients with sarcoma in second line are not enough to cover the use of tyrosine kinase inhibitor.

The aim of the present study was to evaluate the efficacy and safety profile of IE in adult patients with pre-treated advanced STS.

## Methods

This is a retrospective, single centre study conducted at A C Camargo Cancer Center, Sao Paulo, SP, Brazil. Medical records of patients diagnosed with STS and treated with IE between 2010 and 2018 were reviewed. Inclusion criteria were histologic diagnosis of STS, metastatic or locally advanced and irresectable disease, age >18 years old, and available clinical data. Patients with gastrointestinal stromal tumours (GIST), osteosarcoma, chondrosarcoma, Ewing sarcoma, desmoid tumour, rhabdomyosarcoma and desmoplastic small round cell tumour were excluded. This study was approved by the Institutional Ethics Committee (067951/2019).

Patients were treated with etoposide (100 mg/m^2^ for 4 to 5 days) and ifosfamide (1,800 mg/m^2^ for 3 to 5 days) plus mesna (1,800 mg/m^2^ for 3 to 5 days) every 21 days. Dose reductions and use of granulocyte colony-stimulating factor (G-CSF) were implemented according to clinical judgment. Adjuvant and neoadjuvant chemotherapy were considered as first-line treatment if recurrence occurred before 6 months after the end of chemotherapy.

The primary end point was PFS since initiation of IE treatment. Response evaluation (Response Evaluation Criteria In Solid Tumours (RECIST) 1.1) was performed by clinical assessment and imaging studies after 2–3 cycles in the absence of overt progression. Overall disease control rate (DCR) was defined as patients achieving partial response (PR), stable disease (SD) and complete response (CR). PFS was calculated as the time from the first day of treatment with IE to objective tumour progression or death. OS was measured as the time from the start of treatment with IE to death from any cause. Toxicity was evaluated based on Common Terminology Criteria for Adverse Event v4.0 (http://www.eortc.be/services/doc/ctc).

Numerical variables were described with median values. Survivals were estimated by the Kaplan–Meier method and the log-rank test was used to compare the groups. To compare categorical and continuous variables, we used the Chi-square test and Mann–Whitney, respectively. For multivariate analysis and Hazard Ratio determination, when possible, we used the Cox regression model with 95% confidence interval. All *p* values were considered to be statistically significant if <0.05. Statistical analysis was performed with IBM Statistical Package for the Social Sciences 2.0.

## Results

### Patients and treatment characteristics

From January 2010 to December 2018, 33 patients met the inclusion criteria. Median age was 43 years. Majority of patients (36.4%) were diagnosed with leiomyosarcoma (two patients with uterine and ten with non-uterine leiomyosarcoma). Ten patients (28.5%) received two or more previous lines of systemic therapy (Table 1).

Primary tumour resection was performed in 31 patients. Fifteen patients (45.5%) received adjuvant or neoadjuvant chemotherapy (13 and 2 patients, respectively). The most used chemotherapy regimen was the combination of anthracycline and ifosfamide (ten patients), followed by anthracycline alone (four patients, two of them concomitant with radiotherapy) and gemcitabine/docetaxel (one patient). Eighteen patients (54.5%) received adjuvant radiation therapy and 2 (6%) received neoadjuvant radiation. Previous resection of lung metastasis was performed in eight patients, and seven patients underwent resection of local recurrence. No patient received IE as complementary therapy after local treatments for relapsed disease, as well as none underwent local therapy for oligoprogressive disease during IE.

IE was administered in the first line for 5 patients (15.2%), in the second line for 18 (54.5%), in the third line for 8 (24.3%) and in the fourth line for 2 (6%). Anthracycline was previously used in nearly all the patients (94%). The average number of cycles and duration of treatment with IE was 4.3 cycles (range: 1–11) and 83 days (range: 21–305), respectively. Treatment interruption occurred due to disease progression in 19 patients (57.6%), grade 3 or 4 toxicities in 10 (30.3%) and maximum benefit in 4 (12.1%).

### RR and survival

The objective RR during treatment with IE was 9% (three patients had PR; no patient had CR). SD was seen in 17 patients (51.5%). The overall DCR was 60.6%. The progression disease rate was higher in the leiomyosarcoma group as compared to other histologies (58.3% × 28.6%, *p* = 0.14). Table 2 shows the types of response for each histologic subtype.

**Figure 1.**
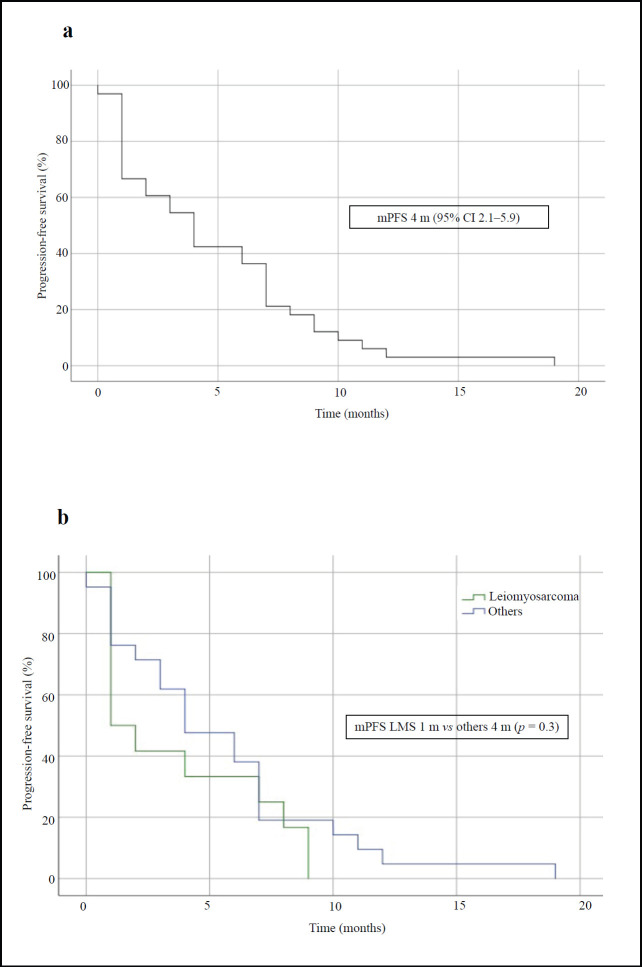
PFS curves of patients treated with IE. (a): Overall population. (b): Patients with leiomyosarcoma comparing with those with other STS. mPFS: median PFS.

**Figure 2.**
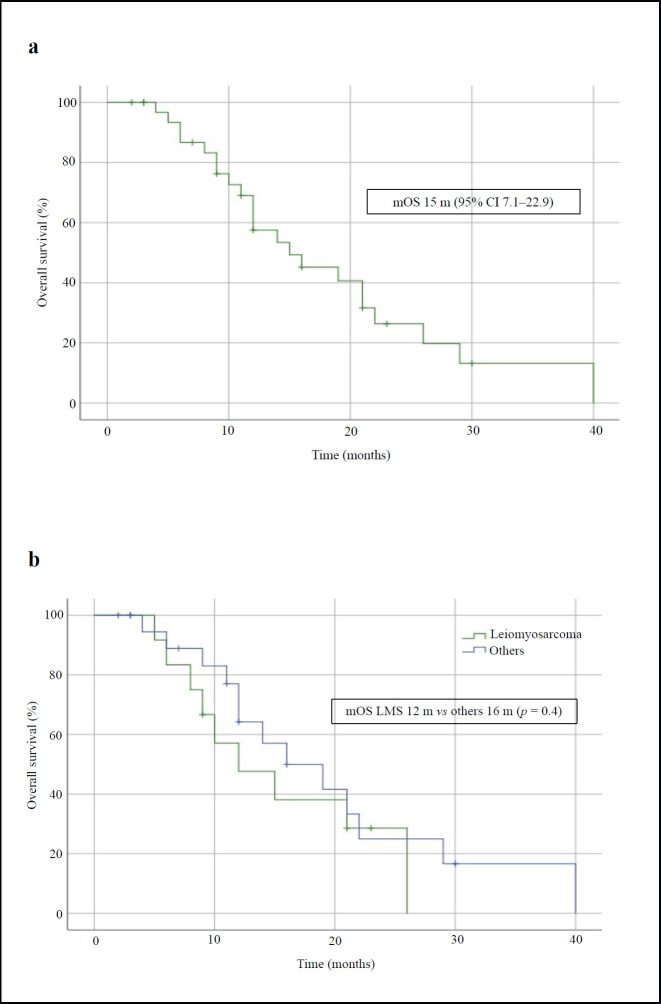
OS curves of patients treated with IE. (a): Overall population. (b): Patients with leiomyosarcoma comparing with those with other STS. mOS: median OS.

After treatment with IE, the median PFS in the overall population was 4 months (95% CI, 2.1–5.9) and the median OS was 15 months (95% CI, 7.1–22.9) – Figures 1a and 2a, respectively. The median PFS for the patients with leiomyosarcoma was 1 month (95% CI, not calculated), and 4 months (95% CI, 1.3–6.7) for the group of the other histologies (*p* = 0.3, Figure 1b). The median OS for patients with leiomyosarcoma was 12 months (95% CI, 4.3–19.7) versus 16 months (95% CI, 7.9–24.1) in the group of the other histologies (*p* = 0.4, Figure 2b).

In the group of patients not experiencing disease progression on IE, the median disease control time after the last cycle was 5 months, ranging from 1.3 to 18.4 months.

On univariate analysis, smoking, treatment line and response to prior chemotherapy were significantly correlated with PFS, and primary tumour surgery was significantly associated with prolonged OS (Table 3).

### Safety and tolerability

Twenty-six patients (78.8%) received G-CSF after each chemotherapy cycle and 19 patients had dose reductions due to myelotoxicity.

The use of IE was at large well tolerated, but ten patients (30.3%) had to discontinue treatment due to toxicity. Among the most serious adverse events (grades 3 and 4), eight patients (24%) presented with febrile neutropenia, three of them with sepsis and admission to the intensive care unit. Blood transfusion was necessary in nine patients (27%) presenting grade 3 anaemia. Two patients had transient grade 3 haematuria, one had transitory grade 3 encephalopathy induced by ifosfamide and one had grade 3 acute kidney injury, which was reversed after chemotherapy interruption. There was no death related to IE treatment.

## Discussion

In this study, we presented a daily practice experience with IE for selected and previously treated patients with STS. Despite modest activity as compared to randomised trials, this regimen showed high toxicity profile. Almost half of the cohort received IE as second-line therapy, and the median OS was 15 months, a finding that is in accordance with other reports [19]. In the Comandone *et al* [20] meta-analysis including ten randomised trials, authors showed that second-line therapy can significantly reduce the risk of progression or death by 49% (HR = 0.51, 95% CI 0.34–0.76, *p* < 0.0008). The pooled median OS was 10.1 for control arms and 13.4 months for experimental arms. Data from randomised phase II and III studies support that exposure to second and further lines of therapy may be responsible for extending survival in patients with metastatic STS. In 2014, the EORTC 62012 trial reported a median OS of 12.8 months in patients treated with single-agent doxorubicin in the first line [8], whereas the 2019 ANNOUNCE trial reported a median OS of 19.7 months in the group of patients receiving this same treatment [6]. This can be attributed, in part, to the development of better histology-guided second-line therapies.

Ifosfamide is an option in the second line for management of certain subtypes of sarcomas, and can reach RRs ranging from 10% to 45% according to dose, administration schedule and histology [21, 22]. But the efficacy of the combination of ifosfamide with etoposide in meta static STS was addressed by few older studies, and comparison between their findings is difficult for many reasons: population selection, number of previous lines of chemotherapy, doses and schedules of the drugs, response evaluation criteria and histologies subtypes included. For example, in the study by Blair *et al* [18], 50% of the cohort was constituted by patients with primary gastrointestinal tract leiomyosarcoma, which would probably be re-classified as GIST in current pathological classification, a well-known chemoresistant tumour. They found an overall response rate (ORR) of 10.5%, and a median OS of 10 months.

In our study, we observed an ORR of only 9%. One-third of the patients received IE in the third and beyond lines of therapy, a scenario in which most sarcomas behave as chemotherapy resistant disease. The other factor that may have impacted our RR is the predominance of leiomyosarcoma in the cohort (36%). Since beginning of last decade, evidence emerged showing that this subtype of sarcoma has limited sensitivity to ifosfamide combinations [23], and based on more recent data [24] the current guidelines consider ifosfamide to be less preferential in the treatment of patients with metastatic non-uterine leiomyosarcoma [3, 25, 26]. Data shown in our study reinforce this, as seen by the higher progression disease rate in the leiomyosarcoma group when compared to the other sarcoma histologies, although not statistically significant (58.3% × 28.6%, *p* = 0.14), with a median PFS of 1 month versus 4 months in the others (*p* = 0.3).

The specific endpoints that best reflect the benefit of systemic therapy in metastatic STS is still debatable. Objective RRs (as defined by a decrease in the size of measurable lesions usually assessed by RECIST) are increasingly seen as poor indicators for evaluating benefit in this heterogeneous group of tumours [27]. A therapeutic agent that is associated with a low objective anti-tumour response can slow the progression of the tumour and prolong survival. Therefore, in clinical practice, the absence of progression is often used as a measure of clinical benefit. We showed a satisfactory DCR with IE, including in a subgroup of patients with leiomyosarcoma.

The median PFS time of second-line agents for metastatic STS is limited. The experimental arm of three randomised, controlled trials with pazopanib [28], eribulin [13] and gemcitabine/dacarbazine [11] resulted in median PFS of 4.6, 3.3 and 4.6 months, respectively. Moreover, the ORR of these trials was not superior to 12% [11, 29]. These data show the need of more effective agents to treat relapsed sarcoma and also the need to conduct trials based on the histology. In our study, patients who received IE in third or fourth lines of therapy presented worst median PFS, an expected result, in part due to a greater resistance to chemotherapy in later lines, as well as to a greater disease burden due to the time elapsed since the initial treatment.

In face of the modest activity of second-line agents, toxicity and quality of life are important to be considered when selecting the treatment regimen [29]. In our cohort, severe neurologic and renal toxicity was observed in one patient each, both reversed after treatment interruption. However, 23% of the patients experienced febrile neutropenia and in most of them G-CSF was administered. Myelosuppression was shown to be the major toxicity of this combination regimen in the previous studies. In the study by Blair *et al* [18], grade 3 and 4 neutropenia occurred in 89% of the individuals. In accordance, myelosuppression was noted in 55.5% of the treatment courses in the study by Yalçin *et al* [30], and one patient died from sepsis due to neutropenia. In addition, in our study, we show that a considerable number of patients (30%) needed to stop treatment due to toxicities. This number is much higher than that observed in other studies evaluating single agent chemotherapy for metastatic STS, in which it ranged between 5% and 14% [12–14]. As a result, considering that the main goal of palliative treatment is to improve quality of life, we cannot neglect that IE could jeopardise quality of life of patients with advanced sarcoma. Furthermore, cost-effectiveness analysis most probably would point that this regimen would be more costly than the other available agents to treat sarcoma in second and third line. Access to the drugs is a barrier that should be discussed individually with patients and the health care providers. Our data support the actions to push official agents to evaluate and approve less toxicity drugs for these patients. One important action is to debate the best options for the patients and make formal statement such as the guidelines for treatment of sarcoma. The recently published Sarcoma European-Latin American Network (SELNET) guideline [26] was reviewed and validated by members from European and Latin American countries.

Our study has several limitations, including the various biases of a retrospective study. The number of individuals is small, and the biological behaviour of sarcomas is largely heterogeneous and singular for each patient. A major weakness of our study is the fact that we could not demonstrate the direct impact of IE in the quality of life of the patients receiving the treatment, which is an important secondary endpoint to be incorporated in the assessment of a therapy in the metastatic setting not just in sarcoma trials. Based solely on the toxicity profile of IE observed in our cohort, we could not recommend this regimen outside a clinical trial.

## Conclusion

In this group of patients with metastatic STS previously treated with doxorubicin, the combination of IE showed PFS and DCR comparable to other studies evaluating second and beyond lines of therapy. Nevertheless, the toxicity profile of the regimen surpass that observed with single agent therapies, and the usage of this regimen in the treatment of most of STS should be discouraged. In regions with limited access to novel agents and/or clinical trials, the best option is to discuss with patient and health care providers alternatives to surpass the barriers to access less toxic agents.

## Conflicts of interest

The authors (LFR, FABC, CAL) declare that there is no conflict of interest with this manuscript.

## Funding statement

No funding received.

## Figures and Tables

**Table 1.** Baseline characteristics of the patients.

Characteristics	*N* (%)
Total	33 (100%)
Gender	
Male	13 (39.4%)
Female	20 (60.6%)
Age, years	
Median (range)	43 (20%–75%)
<60 years	28 (84.8%)
>60 years	5 (15.2%)
ECOG performance status	
0	16 (48.5%)
1	14 (42.4%)
2	3 (9.1%)
Comorbidities^a^	
Yes	15 (45.5%)
No	18 (54.5%)
Smoking history	
Yes	7 (21.2%)
No	26 (78.8%)
Metastasis at diagnosis	
Yes	5 (15.1%)
No	28 (84.9%)
Number of previous lines of chemotherapy	
0^b^	6 (18.2%)
1	17 (51.5%)
2	8 (24.3%)
3	2 (6.0%)
Response to prior chemotherapy^c^	
Responder	16 (48.5%)
Non responder	11 (33.3%)
Histology	
Leiomyosarcoma	12 (36.4%)
Sclerosing epithelioid fibrosarcoma	4 (12.1%)
Myxofibrosarcoma	4 (12.1%)
Undifferentiated pleomorphic sarcoma	4 (12.1%)
Others^d^	9 (27.3%)
Primary site	
Extremities	13 (39.3%)
Abdomen	8 (24.3%)
Thorax	8 (24.3%)
Visceral	4 (12.1%)

ECOG, Eastern Cooperative Oncology Group

a Including hypertension, type 2 diabetes, hypothyroidism, and asthma. One patient had both arrhythmia and heart failure, and another patient had Li Fraumeni syndrome

b All patients received anthracycline-based adjuvant/neoadjuvant chemotherapy

c Not included six patients who received IE as first-line treatment

d Includes liposarcoma (2), spindle cell sarcoma (2), malignant peripheral nerve sheath tumour (2), synovial sarcoma (1), epithelioid sarcoma (1), clear cell sarcoma (1)

**Table 2.** Tumor response according to histologic subtype.

Histology	PR	SD	PD
Leiomyosarcoma (*n* = 12) Non-uterine Uterine	0 0 0	5 4 1	7 6 1
Sclerosing epithelioid fibrosarcoma (*n* = 4)	1	3	0
Myxofibrosarcoma (*n* = 4)	0	3	1
Undifferentiated pleomorphic sarcoma (*n* = 4)	2	1	1
Liposarcoma (*n* = 2)	0	2	0
Spindle cell sarcoma (*n* = 2)	0	1	1
Malignant tumor of the peripheral nerve sheath (*n* = 2)	0	1	1
Synovial sarcoma (*n* = 1)	0	1	0
Epithelioid sarcoma (*n* = 1)	0	0	1
Clear cell sarcoma (*n* = 1)	0	0	1

**Table 3.** Univariate analysis of prognostic factors for PFS and OS in patients treated with IE.

Variable (n)		PFS, months (CI 95%)	*p* value	OS, months (CI 95%)	*p* value
Gender	Male (13)	3.0 (0.0–6.5)	0.85	16.0 (8.4–23.6)	0.64
	Female (20)	4.0 (1.8–6.1)		15.0 (8.7–21.3)	
Age	≤60 years (28)	4.0 (1.4–6.6)	0.72	15.0 (8.9–21.0)	0.96
	>60 years (5)	4.0 (1.8–6.1)		9.0 (NC)	
ECOG performance status	0 (16)	2.0 (0.0–4.6)	0.70	12.0 (7.4–16.6)	0.73
	1 (14)	4.0 (1.4–6.6)		21.0 (14.4–27.6)	
	2 (3)	8.0 (0.0–16.0)		14.0 (NC)	
Smoking	Yes (7)	1.0 (0.1–1.9)	0.002	12.0 (0.0–24.5)	0.52
	No (26)	6.0 (4–7.9)		15.0 (9.3–20.7)	
Primary site	Limbs (13)	4.0 (0.5–7.5)	0.79	14.0 (7.1–20.8)	0.58
	Abdomen (8)	2.0 (0.0–6.2)		19.0 (8.8–29.2)	
	Thorax (8)	3.0 (0.0–8.5)		22.0 (NC)	
	Visceral (4)	1.0 (0.0–4.9)		12.0 (6.9–17.1)	
Primary tumor surgery	Yes (31)	4.0 (2.2–5.8)	0.79	16.0 (8.7–23.3)	0.03
	No (2)	6.0 (NC)		9.0 (NC)	
Prior radiotherapy	Yes (20)	4.0 (2.5–5.4)	0.27	21.0 (8.6–33.4)	0.12
	No (13)	2.0 (0.0–4.0)		12.0 (7.4–16.6)	
Adjuvant or neoadjuvant chemotherapy	Yes (16)	4.0 (0.1–7.9)	0.54	12.0 (10.5–13.5)	0.05
	No (17)	4.0 (2.0–5.9)		21.0 (3.0–28.9)	
Metastasectomy	Yes (15)	6.0 (2.8–9.2)	0.38	21.0 (13.9–28.1)	0.25
	No (18)	2.0 (0.0–4.0)		12.0 (6.6–17.4)	
Treatment line	First line (6)	6.0 (1.2–10.8)	0.02	14.0 (11.5–16.5)	0.21
	Second line (17)	6.0 (3.3–8.7)		19.0 (4.95–33.1)	
	Third line (8)	1.0 (NC)		10.0 (0.0–20.3)	
	Fourth line (2)	4.0 (NC)		12.0 (NC)	
Response to prior chemotherapy	Yes (16)	1.0 (0.0–2.0)	0.03	15.0 (8.5–21.5)	0.67
	No (11)	7.0 (3.1–10.9)		19.0 (11.2–26.7)	

NC, not calculated; ECOG, Eastern Cooperative Oncology Group

## References

[1] Siegel RL, Miller KD, Jemal A (2020). Cancer statistics, 2020. CA: a cancer journal for clinicians.

[2] IARC (2020). WHO Editorial Board WHO classification of tumors: soft tissue and Bone tumors. WHO Classification of Tumours: Soft Tissue and Bone Tumors (2020) 5th Ed 978-92-8324502-5 (IARC).

[3] Casali PG, Abecassis N, Aro HT (2018). Soft tissue and visceral sarcomas: ESMO-EURACAN clinical practice guidelines for diagnosis, treatment and follow-up Ann Oncol.

[4] Gronchi A, Palmerini E, Quagliuolo V (2020). Neoadjuvant chemotherapy in high-risk soft tissue sarcomas: final results of a randomized trial from Italian (ISG), Spanish (GEIS), French (FSG), and Polish (PSG) Sarcoma Groups. J Clin Oncol.

[5] Lyu HG, Haider AH, Landman AB (2019). The opportunities and shortcomings of using big data and national databases for sarcoma research. Cancer.

[6] Tap WD, Wagner AJ, Schöffski P (2020). Effect of doxorubicin plus olaratumab vs doxorubicin plus placebo on survival in patients with advanced soft tissue sarcomas: the announce randomized clinical trial. JAMA.

[7] Spencer RMSSB, Camargo VP, Silva MLG (2020). Brazilian consensus on the diagnosis and treatment of extremities soft tissue sarcomas. J Surg Oncol.

[8] Judson I, Verweij J, Gelderblom H (2014). Doxorubicin alone versus intensified doxorubicin plus ifosfamide for first-line treatment of advanced or metastatic soft-tissue sarcoma: a randomised controlled phase 3 trial. Lancet Oncol.

[9] Santoro A, Tursz T, Mouridsen H (1995). Doxorubicin versus CYVADIC versus doxorubicin plus ifosfamide in first-line treatment of advanced soft tissue sarcomas: a randomized study of the european organization for research and treatment of cancer soft tissue and bone sarcoma group. J Clin Oncol.

[10] Ducoulombier A, Cousin S, Kotecki N (2016). Gemcitabine-based chemotherapy in sarcomas: a systematic review of published trials. Crit Rev Oncol Hematol.

[11] García-Del-Muro X, López-Pousa A, Maurel J (2011). Randomized phase II study comparing gemcitabine plus dacarbazine versus dacarbazine alone in patients with previously treated soft tissue sarcoma: a Spanish group for research on sarcomas study. J Clin Oncol.

[12] Demetri GD, Mehren M, Jones RL (2016). Efficacy and safety of trabectedin or dacarbazine for metastatic liposarcoma or leiomyosarcoma after failure of conventional chemotherapy: results of a phase III randomized multicenter clinical trial. J Clin Oncol.

[13] Schöffski P, Chawla S, Maki RG (2016). Eribulin versus dacarbazine in previously treated patients with advanced liposarcoma or leiomyosarcoma: a randomised, open-label, multicentre, phase 3 trial. Lancet (London, England).

[14] van der Graaf WT, Blay JY, Chawla SP (2012). Pazopanib for metastatic soft-tissue sarcoma (PALETTE): a randomised, double-blind, placebo-controlled phase 3 trial. Lancet (London, England).

[15] Womer RB, West DC, Krailo MD (2012). Randomized controlled trial of interval-compressed chemotherapy for the treatment of localized ewing sarcoma: a report from the children’s oncology group. J Clin Oncol.

[16] Gaspar N, Occean BV, Pacquement H (2018). Results of methotrexate-etoposide-ifosfamide based regimen (M-EI) in osteosarcoma patients included in the french os2006/sarcome-09 study. Eur J Cancer.

[17] Kawai A, Chuman H, Makimoto A (2004). Ifosfamide—etoposide chemotherapy in patients with advanced adult soft tissue sarcomas. J Clin Oncol.

[18] Blair SC, Zalupski MM, Baker LH (1994). Ifosfamide and etoposide in the treatment of advanced soft tissue sarcomas. Am J Clin Oncol.

[19] Italiano A, Mathoulin-Pelissier S, Le Cesne A (2011). Trends in survival for patients with metastatic soft-tissue sarcoma. Cancer.

[20] Comandone A, Petrelli F, Boglione A (2017). Salvage therapy in advanced adult soft tissue sarcoma: a systematic review and meta‐analysis of randomized trials. Oncologist.

[21] Scurr M (2011). Histology-driven chemotherapy in soft tissue sarcomas. Curr Treat Opt Oncol.

[22] Ratan R, Patel SR (2016). Chemotherapy for soft tissue sarcoma. Cancer.

[23] Sleijfer S, Ouali M, van Glabbeke M (2010). Prognostic and predictive factors for outcome to first-line ifosfamide-containing chemotherapy for adult patients with advanced soft tissue sarcomas an exploratory, retrospective analysis on large series from the european organization for research and Tr. Eur J Cancer.

[24] D'Ambrosio L, Touati N, Blay JY (2020). Doxorubicin plus dacarbazine, doxorubicin plus ifosfamide, or doxorubicin alone as a first‐line treatment for advanced leiomyosarcoma: a propensity score matching analysis from the european organization for research and treatment of cancer soft tissue and bone sarcoma group. Cancer.

[25] Garcia del Muro X, Alava E, Artigas V (2016). Clinical practice guidelines for the diagnosis and treatment of patients with soft tissue sarcoma by the spanish group for research in sarcomas (GEIS). Cancer Chemother Pharmacol.

[26] Blay JY, Hindi N, Bollard J (2022). SELNET clinical practice guidelines for soft tissue sarcoma and GIST. Cancer Treat Rev.

[27] Stacchiotti S, Verderio P, Messina A (2012). Tumor response assessment by modified choi criteria in localized high-risk soft tissue sarcoma treated with chemotherapy. Cancer.

[28] Cesne AL, Bauer S, Demetri GD (2019). Safety and efficacy of pazopanib in advanced soft tissue sarcoma: PALETTE (EORTC 62072) subgroup analyses. BMC cancer.

[29] Sharma S, Takyar S, Manson SC (2013). Efficacy and safety of pharmacological interventions in second- or later-line treatment of patients with advanced soft tissue sarcoma: a systematic review. BMC Cancer.

[30] Yalçin S, Güllü I, Barişta I (1998). Treatment of advanced refractory sarcomas with ifosfamide and etoposide combination chemotherapy. Cancer Invest.

